# Association between Dietary Inflammatory Index and Sarcopenia: A Meta-Analysis

**DOI:** 10.3390/nu15010219

**Published:** 2023-01-01

**Authors:** Houze Diao, Feifei Yan, Qingzhen He, Mingyuan Li, Qingzhao Zheng, Qing Zhu, Fang Fang, Weiwei Cui

**Affiliations:** Department of Nutrition and Food Hygiene, School of Public Health, Jilin University, 1163 Xinmin Avenue, Changchun 130021, China

**Keywords:** dietary inflammatory index, sarcopenia, muscle mass, muscle strength, meta-analysis, dose–response relationship

## Abstract

Background: The dietary inflammatory index (DII) is thought to be related to many healthy events. However, the association between the DII and sarcopenia remains unclear. Methods: The meta-analysis was conducted to evaluate the effects of the DII on the risk of sarcopenia utilizing available studies. Up to September 2022, Cochrane, PubMed, Web of Science, Medline, and EMBASE databases were searched to evaluate the relationships between the DII and sarcopenia. A random‒effects model was used to calculate the effect size and 95% confidence intervals (CIs). Result: Eleven studies with 19,954 participants were included in our meta-analysis. The results indicated that a high DII increased the risk of sarcopenia (OR = 1.16, 95%CI [1.06, 1.27], *p* < 0.05). The result of the dose–response analysis showed that the risk of sarcopenia increased by 1.22 times for each 1-point increase in the DII score (OR = 1.22, 95%CI [1.12, 1.33], *p* < 0.05). Conclusion: The meta-analysis demonstrated that the DII is associated with sarcopenia. Considering some limitations in this study, more studies are needed to verify this relationship.

## 1. Introduction

Sarcopenia is induced by a continuous loss in muscle strength, muscle function, and skeletal muscle mass [1]. Relevant research shows that sarcopenia is associated with frailty, falls, functional decline, even severely affecting the quality of life and survival time of the population. Patients with sarcopenia have a decreased ability to maintain daily functions and are more likely to suffer from heart disease, respiratory problems, and cognitive impairments. The average survival time of subjects without sarcopenia is 16.3 years, while subjects with sarcopenia could be reduced to 10.3 years [2]. These phenomena are more common in the elderly. According to the investigation, muscle mass begins to decrease around age of 40, and as much as 15% of the elderly over 65 years old are suffering from sarcopenia, and that figure increases to 50% in the elderly over 80 years old [3]. With the aging of the population, sarcopenia could affect more than 200 million people in the next 40 years. Furthermore, the United States pays more than $18 billion in healthcare costs for sarcopenia each year, and the average increase in healthcare costs for sarcopenia patients is more than $2300 per year. Sarcopenia has posed a major challenge to society and healthcare systems.

As an important part of life, diet is closely associated with the occurrence, development, and rehabilitation of many diseases by regulating the inflammatory state [4]. The DII is formed by fitting the effects of diet on inflammatory markers, including Interleukin-1β(IL-1β), Interleukin 4 (IL-4), Interleukin 6 (IL-6), Interleukin 10 (IL-10), Tumor Necrosis Factor-α (TNF-α), and C-reactive protein (CRP) [5,6,7]. The food frequency questionnaire (FFQs) is the most common method to calculate the DII [8]. Furthermore, the DII divides diet into anti-inflammatory diets and pro-inflammatory diets based on the inflammatory potential score. Briefly, a pro-inflammatory diet which has a higher DII score might lead to increased inflammation in the body, including high amounts of baked goods, fried foods, and fatty meats, whereas an anti-inflammatory diet, which has a lower DII score, might lower the odds of inflammation, including fruits, vegetables, whole grains, lean proteins and diets containing omega-3 fatty acids, whole grains, lean protein, healthful fats, and spices [8,9]. According to relevant studies, inflammatory cytokines may contribute to down-regulate muscle protein and inhibit muscle synthesis by reducing anabolic factors to affect muscle metabolism, thereby increasing the risk of sarcopenia [10,11,12,13].

A healthy diet greatly contributes to the prevention of sarcopenia. However, in the past studies on diet and sarcopenia, people more tended to explore the intake of certain high protein foods and some trace elements. The role of the macro diet pattern on sarcopenia has been ignored. In recent years, the DII as a dietary indicator contained a variety of nutrients and reflected individual dietary patterns. Recently, more and more studies showed that the DII was related to many diseases, such as cardiovascular and cerebrovascular disease, depression, diabetes, cancer, and metabolic syndrome. Similarly, it was reported that there was a relationship between the DII and sarcopenia, and individuals with a higher DII were more likely to develop sarcopenia. However, other studies demonstrated that there was no significant relationship. The effect of the DII on sarcopenia remains unclear, and the relationship has not been demonstrated in a meta-analysis. Therefore, we conducted a meta-analysis of all related observational studies to examine the relationships between the DII and sarcopenia.

## 2. Materials and Methods

### 2.1. Sources and Methods of Data Retrieval

The meta-analysis was carried out in strict accordance with the PRISMA guidelines, and the specific details are shown Table S1 [14]. We searched electronic databases including Cochrane Library, Medline, Web of Science, PubMed, and EMBASE for literature related to the DII and sarcopenia up to September 2022. The following keywords (combined with ‘OR.’ or ‘AND’) were used to search the potential literature on the DII and sarcopenia: dietary inflammatory index, DII, sarcopenia, muscle quantity, muscle mass, muscle function. Language as well as subjects were restricted in the literature search, and only articles published in English and articles with human subjects were included. The specific search details are summarized in Table S2.

### 2.2. Inclusion Criteria

**Figure 1:**
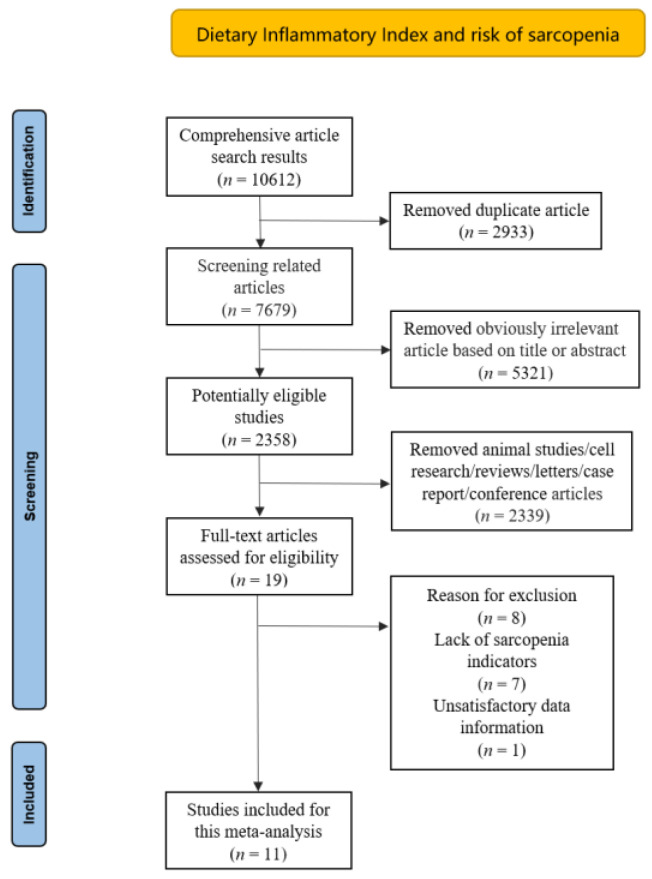
Flow diagram of the literature search and selection.

The inclusion criteria were as follows: (1) the study design was observational studies; (2) the DII was obtained based on a standard calculation method; (3) sarcopenia was defined by local criterion; (4) the literature was based on human subjects; (5) the primary outcomes of interest were presented as odds ratios (ORs) or relative risks (RRs), along with their 95% confidence intervals (CIs), or comprehensive data were provided to calculate them; and (6) duplicate articles, reviews, or conference papers were excluded. Two researchers independently evaluated all of the relevant papers, extracted the final eligible literature, discussed and resolved disagreements with experts in the field (Figure 1).

### 2.3. Data Extraction

We extracted crucial data from the final included research: first author, publication year, region, sample size, sex, mean age of participants, study design, adjusted confounding factors, performance of sarcopenia, DII in different groups, adjusted total effect estimates of sarcopenia, and their corresponding 95% CIs.

### 2.4. Quality Assessment

The bias risk of the observational literature was assessed independently by two investigators by using the Newcastle Ottawa scale (NOS) [15]. This scale consists of three parts: selection, comparability, and exposure, among which each item in the selection and exposure part can obtain at most one point, and the comparability part can obtain at most two points. A quality score greater than 6 was considered to be low bias risk [16]. At the same time, the GRADE system was utilized to evaluate the quality and strength of evidence for included studies [17]. The included trials were divided into four grades: high quality, moderate quality, low quality, and very low quality. Although the initial grade of observational studies was low quality, a higher total effect value, a dose–response relationship, and fewer confounding factors can improve the level of evidence [18].

### 2.5. Statistical Analysis

All statistical analyses were carried out in RevMan version 5.3 and Stata version 12.0 software. The multivariate adjusted effect estimates from all of the included studies were extracted to calculate the pooled estimates and their corresponding 95% CIs via a random effects model. Potential publication bias was evaluated by funnel plot symmetry and Egger’s test. The trim-and-fill method was conducted to correct the result of bias and evaluate the effect of publication bias on the pooled estimates. A statistical heterogeneity analysis was performed by Cochran’s Q statistic and the *I*^2^ statistic. A significant difference was considered to exist among the included studies if the *p* value was < 0.05. The degree of heterogeneity was evaluated according to the *I*^2^ value, in which 25%, 50%, and 75% indicated low, moderate, and high heterogeneity, respectively.

The sources of heterogeneity were explored via a sensitive analysis and subgroup analyses. Sensitive analysis explored whether extreme results affect the pooled risk estimates by excluding each study one by one from all studies. Subgroup analyses were carried out based on sex (male and female), performance of sarcopenia (muscle mass, muscle strength, and physical performance) [19], region of subjects (Asia, Americas, and Oceania), and basic disease (general population and people with basic diseases). Moreover, the method of generalized least squares trend estimation was utilized to explore whether there was a potential dose–response relationship between the DII and sarcopenia. In addition, we applied restricted cubic splines at three knots to seek potential non-linear dose–response relationships.

## 3. Result

A total of 10,612 relevant articles were identified and initially screened from electronic databases according to the search strategy; only 11 articles (19,954 subjects) met our inclusion criteria. The specific details of included studies are summarized in Table 1. Seven of these studies were performed in Asia; three were conducted in Americas; and only one in Oceania. All studies adjusted for covariates that might have an effect on sarcopenia. The adjusted covariates are listed in Table 1. All included studies were published in the last five years. The average value of the NOS score was 8.4 in the assessment of bias risk (all evaluated studies scored greater than 6), indicating a low risk of bias (Table 1 and Table S3). Meanwhile, the GRADE system was utilized to assess the quality of available evidence. The grades of the evidence were considered to be moderate quality (Table 2).

**Figure 2:**
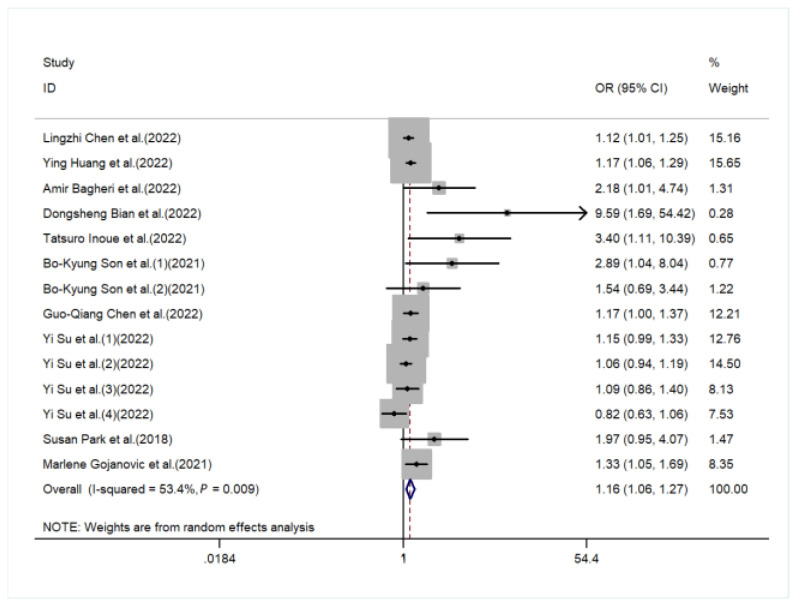
Forest plot of the risk of sarcopenia in subjects with a high DII vs. a low DII [20,21,22,23,24,25,26,27,28,29,30].

The meta-analysis indicated that individuals with a higher DII were more likely to develop sarcopenia compared to a lower DII group (pooled OR = 1.16, 95%CI [1.06, 1.27], *I*^2^ = 53.4%, *p* < 0.05, Figure 2). Although the funnel plot was asymmetric on visual inspection, and a potential publication bias was also found in Egger’s test (*p* = 0.006, Figure S1), the effect size was not significantly changed after the trim-and-fill (OR = 1.12, 95%CI [1.01, 1.25], *p* < 0.05, number of trim and fill = 4), thus indicating that the publication bias had little influence on the above results.

**Figure 3:**
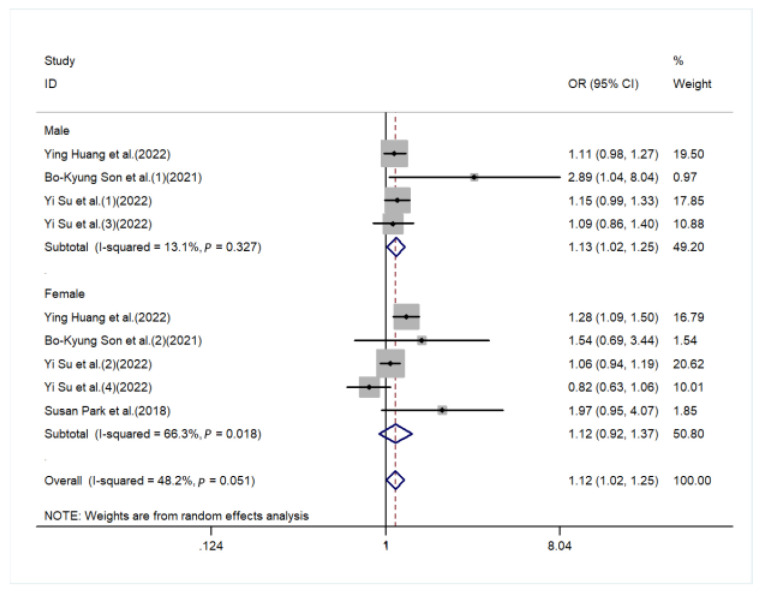
Forest plot for the subgroup of sex [21,25,27,28,29].

**Figure 4:**
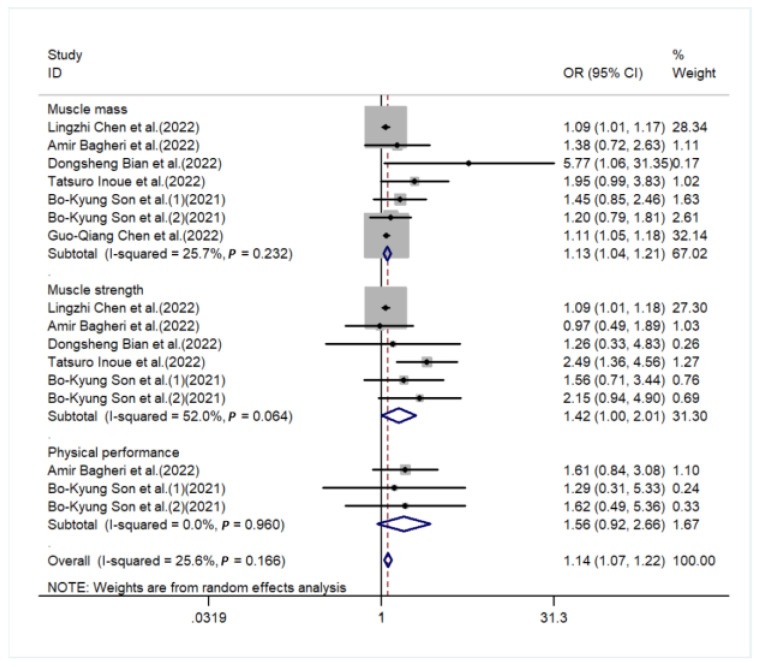
Forest plot for the subgroup of performance of sarcopenia [20,22,23,24,25,26].

**Figure 5:**
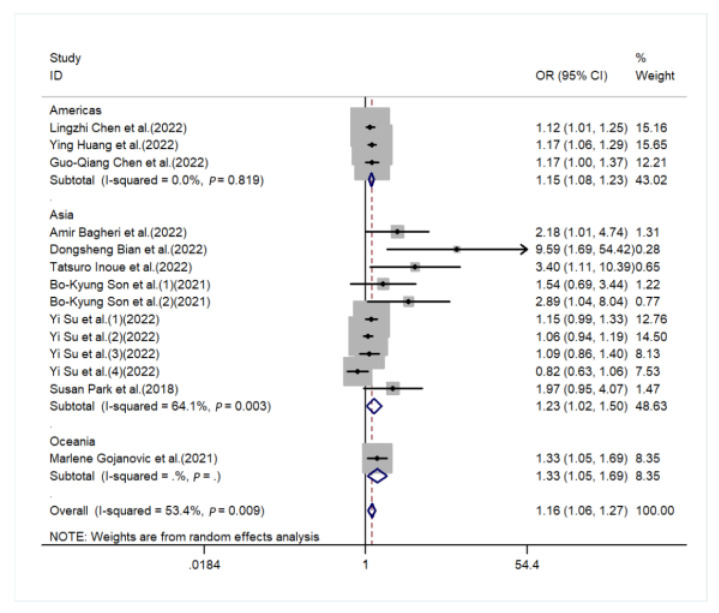
Forest plot for the subgroup of region [20,21,22,23,24,25,26,27,28,29,30].

**Figure 6:**
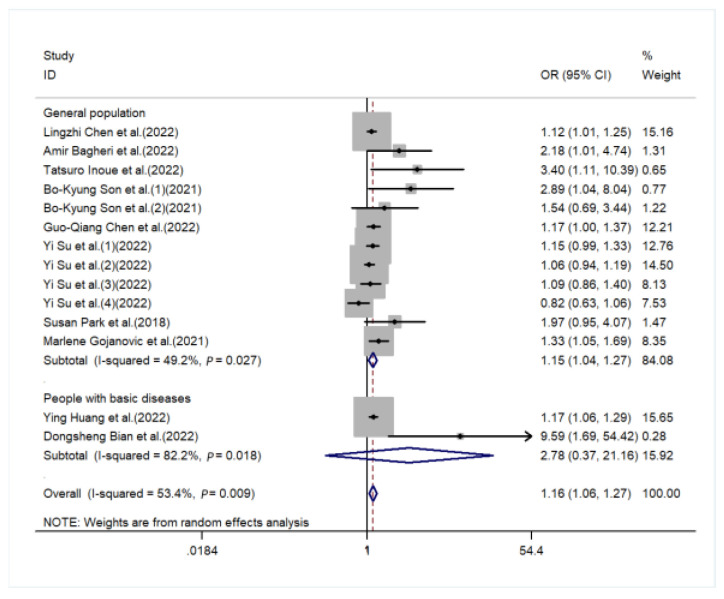
Forest plot for the subgroup of basic disease [20,22,24,25,26,27,28,29,30].

**Figure 7:**
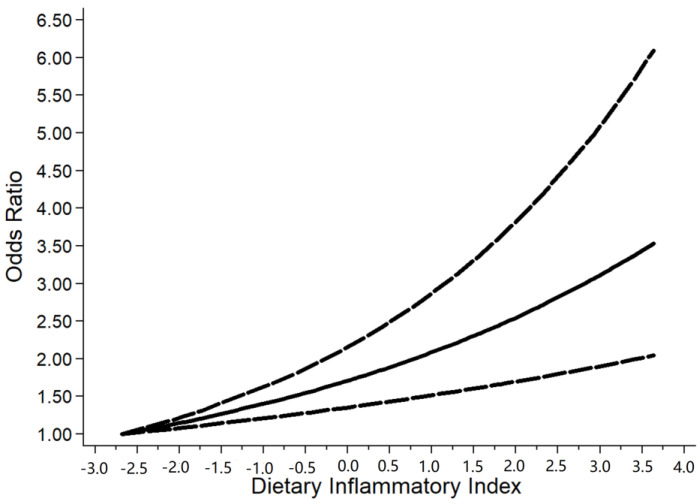
Dose–response plot of the DII and sarcopenia.

The sources of heterogeneity were explored via a sensitive analysis and subgroup analyses. The sensitivity analysis indicated no significant results (Figure S2). Subgroup analyses were carried out in accordance with sex, performance of sarcopenia, region, and basic disease. In the subgroup analysis of sex, the higher the DII score, the higher the risk of sarcopenia in males (OR = 1.13, 95%CI [1.02, 1.25], *p* < 0.05, Figure 3), while a similar trend was not observed in females. In subgroup analyses based on performance of sarcopenia, the DII score was related to sarcopenia in the muscle mass group (OR = 1.13, 95%CI [1.04, 1.21], *p* < 0.05) but not in the muscle strength group or physical performance group (Figure 4). Furthermore, subgroup analyses of all regions showed that the DII was associated with sarcopenia (Figure 5). In the subgroup analyses of basic disease, the DII score was related to sarcopenia in the general population group (OR = 1.15, 95%CI [1.04, 1.27], *p* < 0.05, Figure 6) but not in the people with basic diseases group. The dose–response analysis revealed a potentially linear dose–response relationship between the DII and sarcopenia (*p* > 0.05), with the risk of sarcopenia increasing by 1.22 times for each 1-point increase in the DII score (OR = 1.22, 95%CI [1.12, 1.33], *p* < 0.05, Figure 7).

## 4. Discussion

Due to the increase in the average life expectancy and the aging of the population, sarcopenia is gradually becoming a major public health problem. Dietary factors are known to modulate inflammatory status [4]. The DII is formed by fitting the effects of diet on inflammatory markers including IL-1β, IL-4, IL-6, IL-10, TNF-α, CRP to measure the ability of food promoting inflammation. Meanwhile, inflammation accelerates muscle wasting by triggering protein decomposition and impairing myogenesis [13], thus leading to the occurrence of sarcopenia. A correct understanding of the relationship between the DII and sarcopenia is helpful to prevent sarcopenia. Some studies over the past few years showed that the DII was related with sarcopenia. However, the relationship between the DII and sarcopenia remains controversial. Our meta-analysis showed that the DII score was related to sarcopenia, and the risk of sarcopenia increased by 1.22 times for each 1-point increase in the DII score.

Several generally accepted theories are able to explain the relationship between the DII and sarcopenia. Firstly, the DII was formed by fitting the effects of diet on certain inflammatory cytokines, such as IL-6 and TNF-α. These cytokines increased, representing the occurrence of body inflammation which can lead to impaired skeletal muscle protein synthesis by triggering the ubiquitin–protease system and increasing the risk of sarcopenia [31,32]. Similarly, some epidemiological studies also demonstrated that sarcopenia was affected by inflammatory factors [33,34]. Secondly, some research suggests that chronic inflammation could lead to hypermetabolism and relative anorexia [35], which might increase the risk of sarcopenia. Therefore, in view of the above points, we theorize that a high DII (pro-inflammatory diet) is closely associated with the occurrence of sarcopenia.

The DII was calculated by inflammatory factors. Inflammatory factors, as a direct indicator reflecting the inflammatory state of the body, were closely related to sarcopenia. Based on the results of our meta-analysis, we found that the DII was associated with sarcopenia in males but not in females. This phenomenon might be attributed to the fact that the body of men is generally more sensitive to proinflammatory factors than that of women, and men more likely to have an intense inflammatory response [36]. Therefore, men are more likely to be in an inflammatory state than women, and their prognosis is worse when they are in a state [36]. Relevant studies show that the concentration of plasma inflammatory factors (IL-6 and TNF-α) is associated with the emergence of sarcopenia in men that is more prevalent than that in women, which may be caused by estrogen inhibiting the expression of inflammation-related genes [37,38]. The relationship between serum hypersensitive C-reactive protein levels and sarcopenia was only observed in men [39,40,41]. Similarly, several studies found that sarcopenia was associated with Insulin-like growth factor 1 only in women, which was able to enhance muscle mass and strength, reduce degeneration, inhibit long-term and excessive inflammatory processes [42,43,44]. Furthermore, men tended to consume more pro-inflammatory diets in the elderly stage, which would increase their risk of inflammation, thus affecting the muscle mass and muscle strength [27]. In addition, Cartier’s study showed that obesity might have a protective effect on sarcopenia. The body fat percentages were generally lower for men than for women [45] which might exacerbate the risk of sarcopenia for men [46,47,48]. A recent study showed that in the relationship between the DII and sarcopenia, the men who have higher DII scores were more likely to suffer from depression than the lower DII group. Among women, those with a higher DII had lower physical activity, poor social skills, and a poor oral environment. Men and women may have different relationships between the DII and sarcopenia as a result of these phenomena [25]. A better understanding of the mechanism of sex on the relationship between the DII and sarcopenia is required.

Currently, the performance of sarcopenia includes muscle mass, muscle strength, and physical performance [19]. Our subgroup analyses showed that a high DII score was bound up with decreased muscle mass but not with the other two performances. This difference might be due to the fact that muscle mass is directly related to muscles, while muscle strength and physical performance are affected by many other organism factors, such as bones, balance, and other nerve conduction. Therefore, the sensitivity of the other two performances is lower than muscle mass. Furthermore, this difference might be due to their different measurement methods. Muscle mass was measured by imaging technology (CT scan, MRI, and DXA) [49,50,51], which would give an accurate and direct indication of muscle condition. Nevertheless, muscle strength and physical performance were measured by some relatively subjective indicators (handgrip strength and usual gait speed), which are more easily affected by other factors [52]. According to the diagnostic criteria for sarcopenia [19], sarcopenia could be divided into three stages: presarcopenia, sarcopenia, and severe sarcopenia. In the stage of presarcopenia, only the standard of muscle mass reduction is required; there is no need to meet the other two criteria. In the stage of sarcopenia, the reduction in muscle mass is a necessary condition, and the reduction in the other two criteria can meet either of them. The stage of severe sarcopenia is a criterion for all three. Most of the patients with sarcopenia we included were in the stage of sarcopenia, so muscle mass reduction is significant, and the decrease in muscle strength and physical performance cannot be significant. Furthermore, some studies also showed that the DII was associated with muscle mass [20,25] but not with muscle strength or physical performance [11,22,53]. The results of this study are in accordance with our findings. Meanwhile, limited by the number of studies, more studies are needed to explore its specific mechanism in the future.

It can be seen from the results that pro-inflammatory diets will increase the risk of sarcopenia, while anti-inflammatory diets will decrease the risk of sarcopenia. This provides us with a macro dietary direction to prevent and control sarcopenia, which reduces the total incidence of sarcopenia in the population by increasing the intake of anti-inflammatory diets. At the same time, a mediation analysis [26] showed that the DII and depressive symptoms are related to sarcopenia. Symptoms of depression play a significant mediating role on the related manifestations of the DII and sarcopenia (muscle strength, muscle mass, and physical performance). When the DII is used to prevent and manage sarcopenia, it provides a new supplementary direction. It is necessary to take comprehensive management measures to the mental health of patients with sarcopenia and further reduce the incidence rate of sarcopenia.

Some of the strengths of this study are as follows. Firstly, to our knowledge, this is the first meta-analysis article to explore the relevance between the DII and sarcopenia. Secondly, we used the dose–response relationship to explore the linear relationship between the DII and sarcopenia. Furthermore, we divided sarcopenia into three performances to explore the relationship between the DII and sarcopenia further. Finally, the quality of the outcomes we included in the article was high, which ensured the stability of the association between the DII and sarcopenia. However, this meta-analysis had some limitations. Firstly, these were observational studies included in the meta-analysis; their hierarchy of evidence is lower than that of randomized controlled trials. Secondly, food frequency questionnaires were used to collect DII scores for the whole studies and based on self-reports, which may cause recall bias. Additionally, the measures of the DII score and diagnostic standard for sarcopenia were not be unified in all studies, so the relationship between the DII and sarcopenia might be affected. Finally, a limited number of studies (only 11 studies) was included in the article, and the sample sizes of the contained studies varied widely. Therefore, it is necessary to conduct large-scale prospective cohort studies and basic research to obtain more conclusive evidence.

## 5. Conclusions

In summary, the meta-analysis indicated that the DII is associated with sarcopenia. Meanwhile, the result of the dose–response analysis showed that sarcopenia increased by 1.22 times for each 1-point increase in the DII score. In light of the limitations observed in this meta-analysis, more studies are needed to verify the relationship.

## Figures and Tables

**Table 1:** Characteristics of the included observational studies.

Study	Year	Region	Design	Participant Characteristics	Sample Size	Age	Male (%)	DII Analysis	Diagnosis of Sarcopenia	Variables Adjusted	NOS
Ling zhi Chen et al.	2022	United States	cross sectional	non-institutionalized community-dwelling residents in the United States	1863	62.1	49.00	Categorized (T3:T1) Continuous	A combination of low muscle mass and low muscle strength was used as a representation of sarcopenia	age (years), sex, race, education, marital status, nativity, smoking, physical activity level and BMI, Chronic disease, Energy, and Protein	9
Ying Huang et al.	2022	United States	cross sectional	CKD participants with complete data for DII and sarcopenia in the United States	2569	55.6	45.11	Categorized (T3:T1) Continuous	FNIH	age, gender, race, income, physical activity, smoking, alcohol drinking, diabetes, hypertension, overweight, central obesity, dyslipidemia, cancer, arthritis, heart disease, eGFR, ACR, hypoalbuminemia, low energy intake, low protein intake, CRP, WBC, NLR, and NHANES strata	8
Amir Bagheri et al.	2022	Tehran, Iran	cross sectional	represents the older population in the district 6 of Tehran (age > 55)	300	66.8	50.00	Categorized (T3:T1)	EWGSOP	age, sex, energy intake, physical activity, smoking, alcohol consumption, medication use, and positive history of disease	9
Dongsheng Bian et al.	2022	China	cross sectional	CD patients who received anti-TNF-α therapy at Shanghai Rui jin Hospital	140	32.5	72.14	Categorized (T4:T1)	(AWGS) 2019	sex, age, BMI, smoking status, alcohol consumption, nutritional status, disease activity, total energy intake, disease duration, Montreal Classification, Location	7
Tatsuro Inoue et al.	2022	Japan	cross sectional	ambulatory patients aged 65 years or older in Japan	304	77.6	32.60	Categorized (T4:T1)	(AWGS) 2019	age, sex, comorbidity, BMI, physical activity	7
Bo-Kyung Son et al.	2021	Japan	cross sectional	community-dwelling older adults in Japan	1254	74.6	51.80	Categorized (T3:T1)	(AWGS) 2019	age, education level, protein intake, physical activity, medical history, eating alone, Lubben Social Network Scale (LSNS) social ties	9
Guo-Qiang Chen et al.	2022	United States	cross sectional	non-institutionalized community-dwelling residents in the United States	6082	37.2	51.99	Categorized	FNIH	age, sex, race, educational level, marriage status, family poverty income ratio, smoking status, drinking status, physical activity level, BMI status, diabetes, and hypertension	9
Yi Su et al. (a)	2022	China	prospective cohort	Chinese people aged ≥65 years were recruited from local communities in Hong Kong (follow up for 14 years)	2292	72.5	50.01	Categorized (T3:T1)	(AWGS) 2019	age, BMI, current smoking, previous fracture, hypertension, diabetes, chronic obstructive lung disease, cardiovascular disease, rheumatoid arthritis, nonsteroidal anti-inflammatory agent use, osteoporosis medication, vitamin D status, and physical activity level	8
Yi Su et al. (b)	2022	China	prospective cohort	Chinese people aged ≥65 years were recruited from local communities in Hong Kong (follow up for 4 years)	2997	72.0	47.51	Categorized (T3:T1)	(AWGS) 2019	age, BMI, estimated glomerular filtration rate, current smoking, physical activity level, previous fracture, hypertension, diabetes, chronic obstructive lung disease, cardiovascular disease, rheumatoid arthritis, nonsteroidal anti-inflammatory agent use, and osteoporosis medication	8
Susan Park et al.	2018	Korean	cross section	Korean postmenopausal women over 50 years old	1344	62.3	-	Categorized	Sarcopenia was diagnosed if the percentage-applied value acquired by dividing appendicular skeletal muscle mass (ASM) by weight	age, family income, regular exercise, education status, smoking, and female hormone supplements	9
Marlene Gojanovic et al.	2021	Australian	cross section	Individuals aged 60 years and over in Australian	809	66.4	65.60	Continuous	(AWGS) 2019	race, sex, and body fat percentage, sex×age interaction term	9

NOS, Newcastle–Ottawa scale; BMI, Body mass index; CKD, chronic kidney disease; FNIH, BMI-adjusted ALM (ALM_BMI_): men were judged as sarcopenia if ALMBMI < 0.789, and women < 0.512; eGFR, estimate Glomerular Filtration Rate; ACR, urine Albumin to Creatinine Ratio; CRP, C Reactive Protein; WBC, White Blood Cell; NLR, Neutrophil-Lymphocyte Ratio; EWGSOP, the combination of both low muscle mass and low muscle function; CD, Crohn’s disease; (AWGS) 2019, Low muscle mass (ASMI < 7.0 kg/m^2^ for male, ASMI < 5.7 kg/m^2^ for female, via BIA) and low handgrip strength (handgrip strength < 28 kg for male, handgrip strength < 18 kg for female) were identified as sarcopenia.

**Table 2:** The Summary of Findings (SoF) with GRADE system.

Risk of Sarcopenia with Different Dietary Inflammatory Index Levels			
Population: Subjects with sarcopenia vs. normal subjects			
Settings: Seven studies were conducted in Asia; three studies were conducted in the United States; one study was conducted in Australia			
Trials: Subjects with sarcopenia			
Controls: Normal subjects			
Outcomes	OR (95% CI) ^a^	No of participants (studies)	Quality of the evidence Comments (GRADE)
sarcopenia	1.16 (1.06,1.27)	19,954 (eleven studies)	⊕⊕⊕ MODERATE ^b^
GRADE working group grades of evidence High quality: We are very confident that the true effect lies close to that of the estimate of the effect Moderate quality: We are moderately confident in the effect estimate: The true effect is likely to be close to the estimate of the effect, but there is a possibility that it is substantially different Low quality: Our confidence in the effect estimate is limited: The true effect may be substantially different from the estimate of the effect Very low quality: We have very little confidence in the effect estimate: The true effect is likely to be substantially different from the estimate of effect Abbreviations: OR, odds ratio; CI, confidence interval			

^a^ Results for dietary inflammatory index levels of subjects with sarcopenia compared with controls. ^b^ Upgraded by one level due to a dose‒response relationship between sarcopenia and dietary inflammatory index (The higher dietary inflammatory index, the higher risk of sarcopenia).

## Data Availability

Data from published articles.

## Supplementary Materials

The following supporting information can be downloaded at: https://www.mdpi.com/2072-6643/11/2/338/s1. Figure S1: Funnel plot for the effect estimates of the DII. Figure S2: Sensitivity analysis for included studies. Table S1: PRISMA 2020 Checklist. Table S2: The specific search strategies. Table S3: The risk of bias by the Newcastle-Ottawa scale (NOS).
